# Supplementary material for: Process Engineering of Biopharmaceutical Production in Moss Bioreactors via Model-Based Description and Evaluation of Phytohormone Impact
Source: Front Bioeng Biotechnol. 2022 Feb 17;10:837965. doi: 10.3389/fbioe.2022.837965 (PMC8891706; doi:10.3389/fbioe.2022.837965)
Supplement: Supplementary file 1 [file DataSheet1.doc]

***Supplementary Material***

**Supplementary Table S1**. Primers used for qPCR and qRT-PCR to analyze number of expression cassettes integrated in the genome and expression levels of the transgene, the auxin responsive gene *PpIAA1A*, and *actin* gene *PpAct7a*. The gene model used for the prediction of the sequence is mentioned between brackets.

| **Target gene** | **Primer forward** | **Primer reverse** |
| --- | --- | --- |
| ***MFHR1* gene (for copy number and expression level)** | TGGATGGACCAATGATATTCC | ATTCCCGATCTGGTTCCATT |
| ***PpAct7a* (Pp3c3_33410V3.1)** | TGCAGCACGGTGTATCTCTC | GGCACCAGCGCTAAACAG |
| ***PpIAA1A* (Pp3c8_14720V3.1)** | CCGCAAAAGTCTAGTGAGCA | ACAGTCGTTGCTGGCATTC |
| ***L21* (Pp3c13_2360.V3.1)** | CCTGCGAGGTTCCCGTAA | TGCTTGTTCATCACGACACCA |
| ***EF1*-α (Pp3c2_10310V3.1)** | CGACGCCCCTGGACATC | CCTGCGAGGTTCCCGTAA |
| **5’-HR (*PpAct5* promoter)** | GCTTGGTCCGAGGCTATTATT | TAGGTGCAACCCGCTGTT |
| ***PpCLF*_primers 1**  **(Pp3c22_22940V3.1)** | AGCAATGTCCGTGCCTACTT | TTGTAAGAATCACTCACCCACAG |
| ***PpCLF*_primers 2 (Pp3c22_22940V3.1)** | GTATTGGCGATCCCACTCTT | GCATAAAATAGGTCACAGATTGAGG |
| ***Hpt* selection cassette** | ATACGAGGTCGCCAACATCT | TGCCTCCGCTCGAAGTAG |

**MGASRSVRLA FFLVVLVVLA ALAEAEATFC DFPKINHGIL YDEEKYKPFS QVPTGEVFYY** 60 **SCEYNFVSPS KSFWTRITCT EEGWSPTPKC LRLCFFPFVE NGHSESSGQT HLEGDTVQII** 120 **CNTGYRLQNN ENNISCVERG WSTPPKC*RSE* *D*CNELPPRRN TEILTGSWSD QTYPEGTQAI** 180 **YKCRPGYRSL GNIIMVCRKG EWVALNPLRK CQKRPCGHPG DTPFGTFTLT GGNVFEYGVK** 240 **AVYTCNEGYQ LLGEINYREC DTDGWTNDIP ICEVVKCLPV TAPENGKIVS SAMEPDREYH** 300 **FGQAVRFVCN SGYKIEGDEE MHCSDDGFWS KEKPKCVEIS CKSPDVINGS PISQKIIYKE** 360 **NERFQYKCNM GYEYSERGDA VCTESGWRPL PSC*EDSTGK*C GPPPPIDNGD ITSFPLSVYA** 420 **PASSVEYQCQ NLYQLEGNKR ITCRNGQWSE PPKCLHPCVI SREIMENYNI ALRWTAKQKL** 480 **YSRTGESVEF VCKRGYRLSS RSHTLRTTCW DGKLEYPTCA KRVDHHHHHH HH** 532

**Supplementary Figure S1.** Amino acid sequence of MFHR1 produced in Physcomitrella as case study. The signal peptide is shown in red, the SCRs 1-2 of human factor H- related protein 1 (FHR1) are shown in blue, the SCRs 1-4 and 19-20 of human factor H are shown in orange and green, respectively, and linkers in italics. The polymorphism V62I is highlighted.


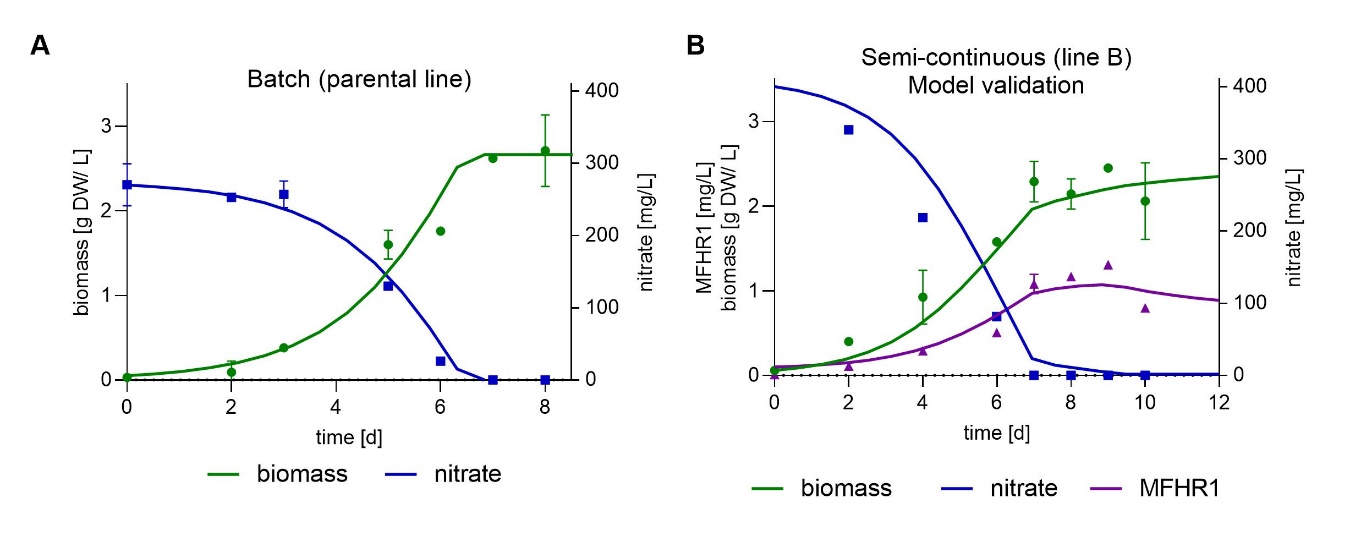


**Supplementary Figure S2.** Mathematical modelling and simulation to describe the growth kinetics of Physcomitrella and the production of the recombinant protein MFHR1. (**A**) parental line (*Δxt/ft*) (**B**) Validation of the kinetic model using semi-continuous operation mode under different conditions. The operation started at day 7 with D= 0.2 d^-1^. Estimated parameters listed on table 1 were used. The solid lines represent modelled data; experimental data are shown with symbols. Parameters were estimated using differential evolution (DE) implemented in Scipy in Python 3.6. Data represent mean ± standard deviations (SD) from two measurements.


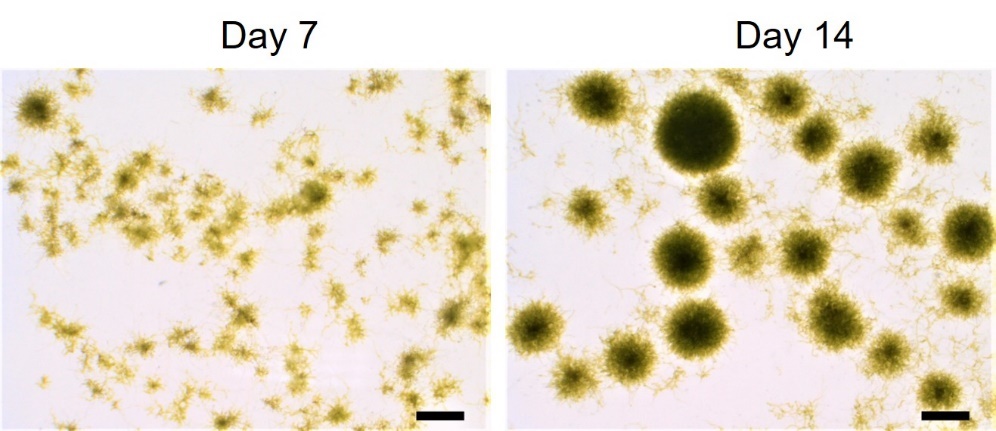


**Supplementary Figure S3**. Light-microscopic photographs of moss-suspension cultures in stirred-tank bioreactor operated in semi-continuous mode. Big pellets are observed at the end of the operation. Scale bar: 1 mm


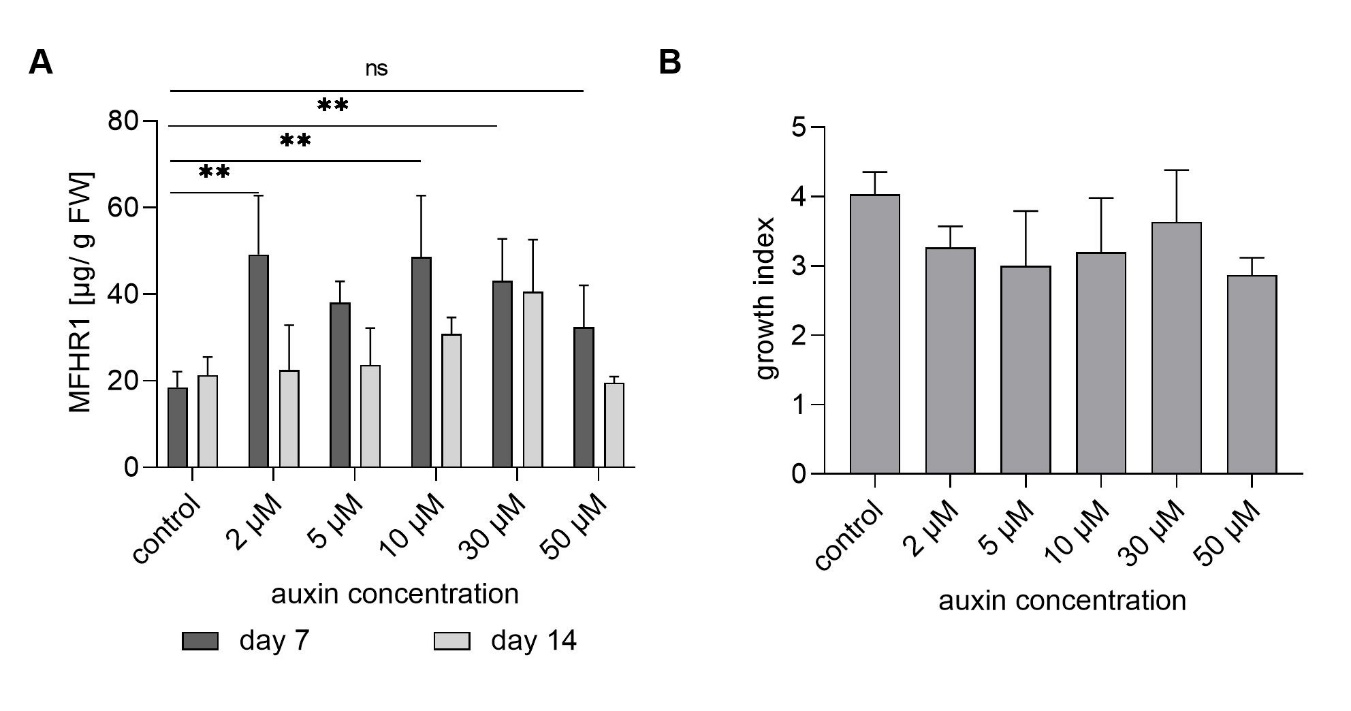


**Supplementary Figure S4.** Effect of NAA concentration on specific MFHR1 production **(A)** There were no significant differences in MFHR1 productivity upon NAA treatment at different concentration (2- 50 µM) at shaken-flask scale (One-way ANOVA, Bonferroni Post-hoc). Significant differences compared to the control without NAA are shown at day 7 (One-way ANOVA, Dunnet Post-hoc). Data represent mean values ± SD from three biological replicates.  **(B)** Growth index of transgenic moss line A cultivated in shaken flasks under different NAA concentrations. Data represent mean values ± SD from three biological replicates.

**
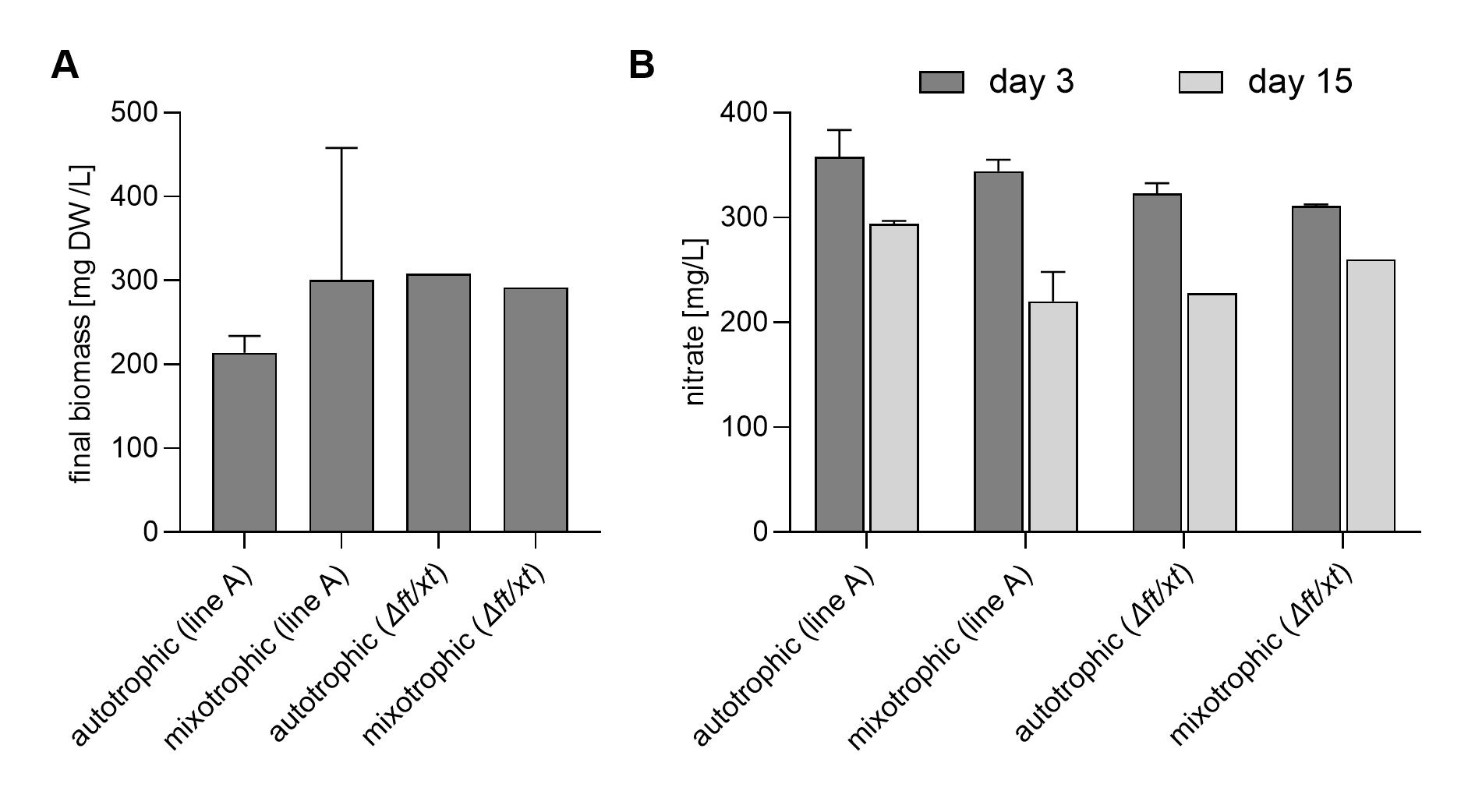
**

**Supplementary Figure S5.** Effect of sugar addition on the growth of Physcomitrella. **(A)** Final biomass after 15 days. The initial cell density was 100 mg DW/L **(B)** Nitrate uptake at shaken flask scale under autotrophic and mixotrophic (1% sucrose and 50-70 µmol/m^2^s light, photoperiod 16/8 h) conditions. Moss transgenic line A and the parental line *Δ ft/xt* were used. Data represent mean values ± SD from three biological replicates.


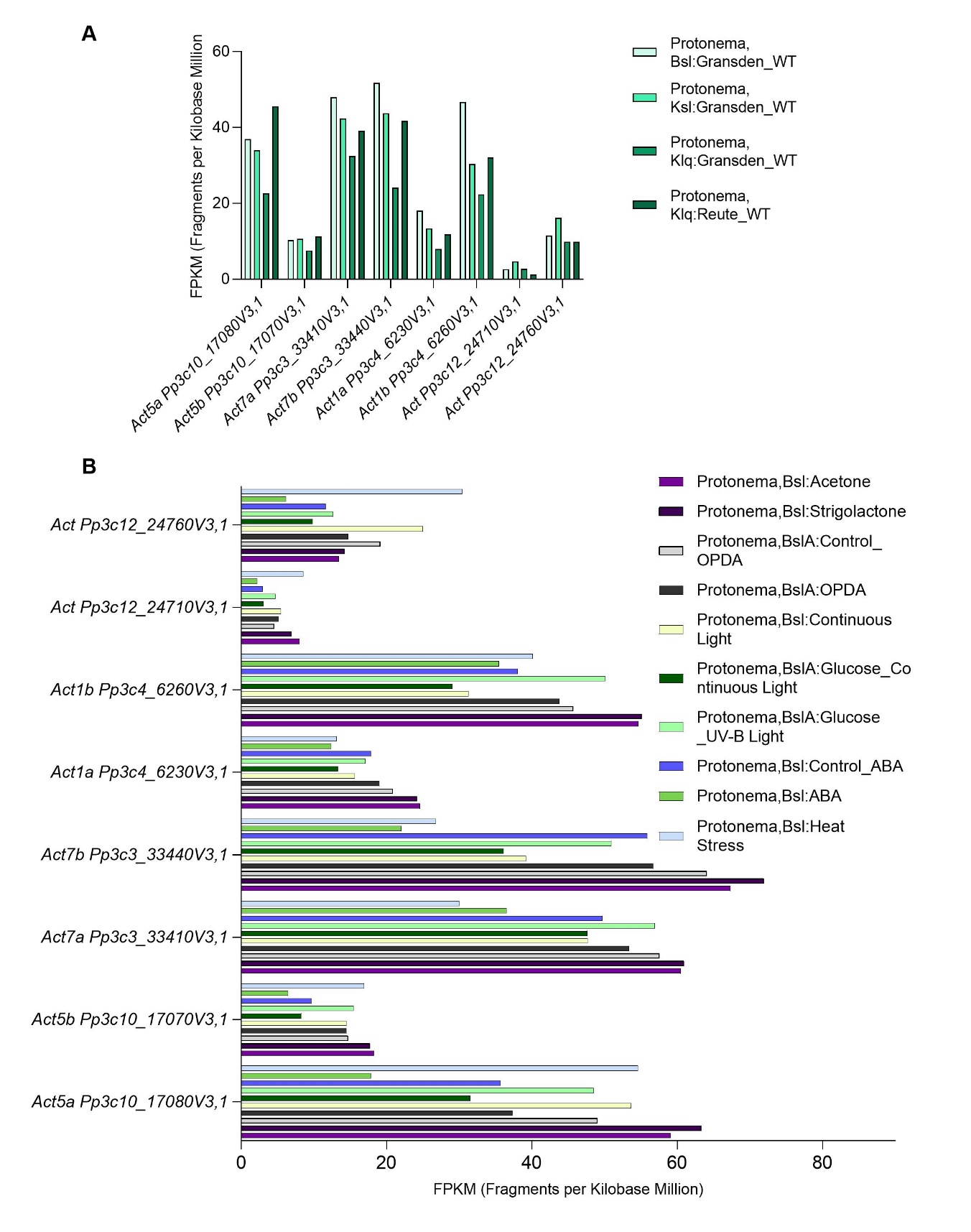


**Supplementary Figure S6.** Actin genes expression levels in protonema of Physcomitrella obtained from PEATmoss (Fernandez-Pozo *et al.*, 2020)**.** (**A**) Expression in Reute and Gransden ecotypes. Dataset “RNAseq developmental stages” was used. (**B**) Expression in protonema under different conditions. Dataset “RNAseq protonema treatments” was used. The abbreviations describe the culture medium, Bsl = BCD solid, BslA = BCDA (ammonium), Klq = Knop liquid, Ksl = Knop solid.


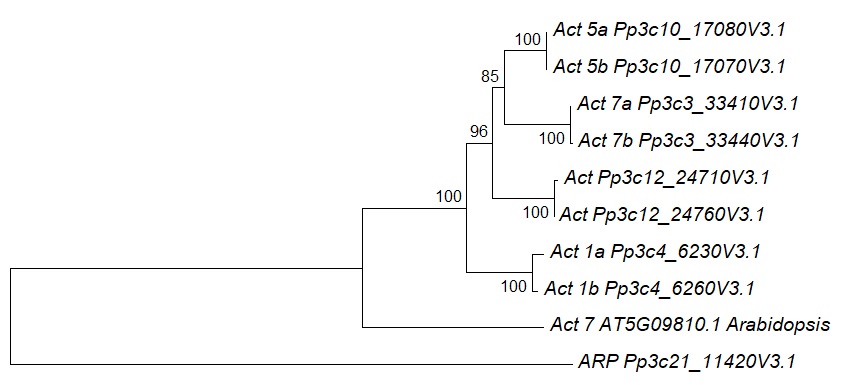


**Supplementary Figure S7.** Neighbor-Joining dendrogram of *actin* genes (CDS) from *P. patens* and *Actin 7* from *A. thaliana*. The evolutionary distances were computed using the Kimura 2-parameter method. Branch support was provided by bootstrap resampling (10000 replicates). As outgroup an actin related protein (ARP) was included. The dendrogram was built using MEGA5. *Act 7b* Pp3C3_33440V3.1 was called *Act 3* by Weise *et al.*, (2006).


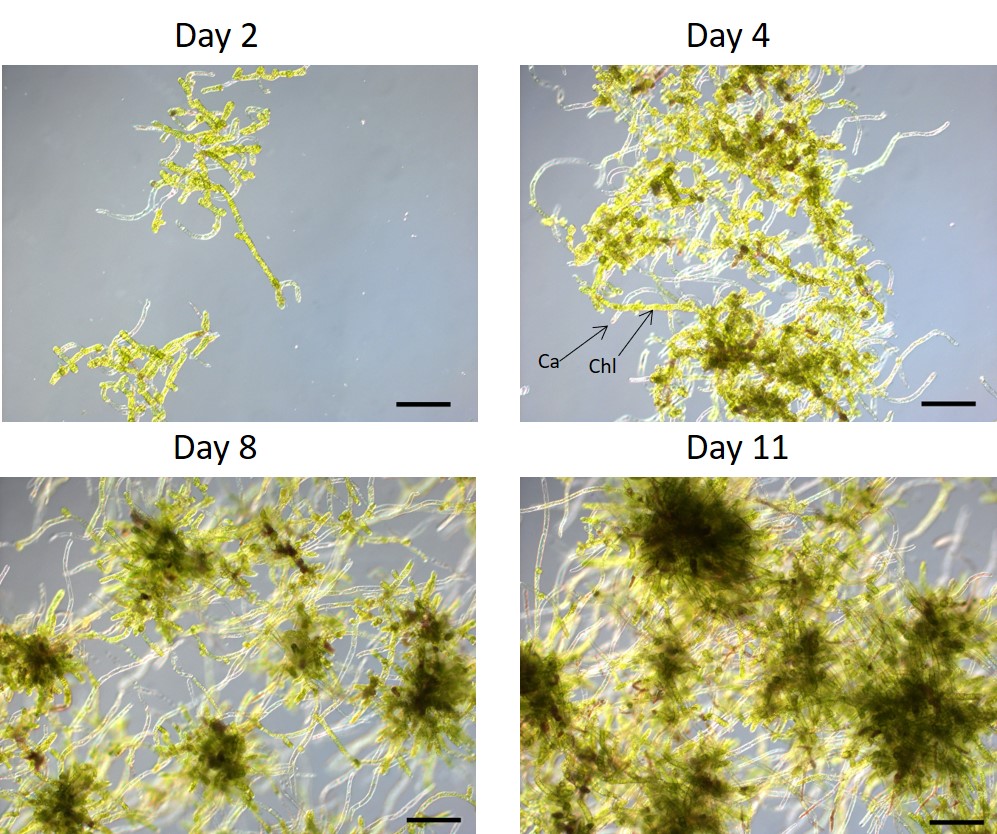


**Supplementary Figure S8.** Representative light-microscopic images of Physcomitrella protonema cultivated in 5L stirred-tank bioreactor in batch operation mode with NAA (10 µM) supplementation at day 3. Scale bars: 200 µm. Chloronema predominates the first 2 days, tissue differentiation to caulonema occurs and pellets are visible after 9 days approximately. Chloronema (Chl) and caulonema (Ca) are marked by arrows at day 4.

## Supplementary methods

**Light distribution in the bioreactor and specific growth rate (light as substrate). Methodology described by Evers (1991) (Supplementary eq 1-5).** l_0_ is the incident light [µmol/(m^2^s)], σ_x_ is the cell absorption coefficient [m^2^/g] and r_R_ is the cylinder radius [m]. The path length of light (p) is function of a distance from vessel surface (L) and angle of light path (ϴ). I(L,c_x_) is the mean light intensity as a function of biomass concentration (c_x_) and location in the vessel.
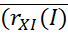
) is the overall biomass growth rate considering the light as the limiting substrate.


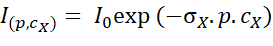
 (Supplementary eq 1)


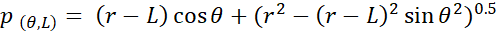
 (Supplementary eq 2)


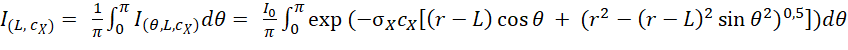


(Supplementary eq 3)


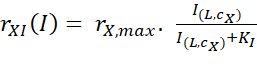
 (Supplementary eq 4)


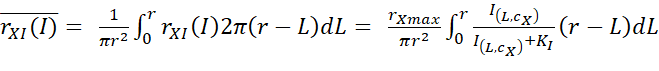


(Supplementary eq 5)
